# Evaluation of application potential of dye-decolorizing peroxidase from *Bacillus amyloliquefaciens* in bioremediation of paper and pulp mill effluent

**DOI:** 10.3389/fmicb.2022.1031853

**Published:** 2022-10-21

**Authors:** Jing Ren, Xiaodan Li, Weitao Zhang, Zhuofan Li, Quan Wang, Shuna Li, Shuxiang Wang, Hongya Li

**Affiliations:** ^1^College of Life Sciences, Hebei Agricultural University, Baoding, China; ^2^Hebei Animal Husbandry General Station, Shijiazhuang, Hebei, China; ^3^Hebei Forage Microbial Technology Innovation Center, Baoding, Hebei, China

**Keywords:** dye-decolorizing peroxidase, *Bacillus amyloliquefaciens*, substrate profile, biological treatment, phytotoxicity, cytotoxicity, paper industrial effluent

## Abstract

Pulp and paper mill effluent is rich in recalcitrant and toxic pollutants compounds and causes pollution. To find an efficient biocatalyst for the treatment of effluent, a dye-decolorizing peroxidase from *Bacillus amyloliquefaciens* MN-13, which is capable of degrading lignin, was used for the bioremediation of paper and pulp mill effluent. The dye-decolorizing peroxidase from *Bacillus amyloliquefaciens* (BaDyP) exhibited high-redox potential to 2, 2′-azinobis (3-ethylbenzothiazoline- 6-sulfonic acid) ammonium salt (ABTS), veratryl alcohol, Mn^2+^, reactive blue 19, reactive black 5 and lignin dimer guaiacylglycerol-beta-guaiacyl ether (GGE). When GGE was used as substrate, BaDyP broke β-O-4 bond of GGE and then oxidize Cα to generate vanillin. The *K_m_* values for ABTS and veratryl alcohol were 2.19 mm and 0.07 mm, respectively. The *V_max_* for ABTS and veratryl alcohol were 1.8 mm/min and 14.12 mm/min, respectively. The BaDyP-mediated treatment of pulp and paper mill effluent led to significant reduction of chemical oxygen demand (COD) and color. When 5% (v/v) of effluent was treated with BaDyP for 12 h at 30°C and pH 2, the removal of COD, color, and lignin was achieved at 82.7, 80.2, and 78.20%, respectively. In detoxification assay, the seeds of *Vigna unguiculata* grown in treated effluent showed a significant increase in germination rate from 66.7% (untreated effluent) to 90%, and in radicle length from 0.68 cm (untreated effluent) to 1.26 cm, respectively. In the meanwhile, the inhibition of *Escherichia coli* and *Bacillus subtilis* by the treated effluent reduced significantly as compared to untreated effluent, indicating high detoxification performance of BaDyP for the treatment of pulp and paper mill effluent. The findings suggest that BaDyP is a potential catalyst for bioremediation of pulp and paper mill effluent, as it is effective in substantial lowering of pollutants load as well as reduces COD, color, and toxicity of effluent.

## Introduction

Pulp and paper industry is one of the most important industrial sections in the world. For a long time, pulp and paper mills have been facing challenges of water-consuming and environmental pollution (M'Hamdi et al., 2017). It is estimated that approximately 60 m^3^ effluent/ton of paper is produced in the paper manufacturing process (Bakraoui et al., 2020). Pulping and bleaching are considered as the two important processes and the sources of most of the pollution involved in paper manufacturing. Pulping is a complex manufacturing process that involves chemical and mechanical treatment of various plant material, in which lignin and hemicelluloses are separated out of plant and released into effluent as waste (Tripathy, 2020). Bleaching is a multi-step chemical treatment of pulp to increase brightness accompanied with various compounds, such as chlorine, hydrogen peroxide, ozone, etc. (Chaudhry and Paliwal, 2018). As a result, the final effluents generated from paper and pulp mill are potentially very polluting, in which a considerable amount of pollutants were characterized with dark color, high organic content, high biochemical oxygen demand (BOD), and chemical oxygen demand (COD; Hossain and Ismail, 2015; Costa et al., 2017). The environmental impact of paper mill effluents depends not only on their chemical nature, but also on their dark coloration that affects negatively aquatic fauna and flora (Haq and Raj, 2020). The primary contributors to the dark color and toxicity of wastewater are lignin and its chlorinated derivatives (e.g., lignosulfonic acid, resins, phenols, and hydrocarbons), which are resistant to removal by the primary sedimentation. Therefore, it is crucial to reduce and/or remove pollution load before being discharged into the environment.

Recently, several physicochemical approaches have been established for the treatment of paper mill effluents, *e. g.,* sedimentation and floatation, coagulation and precipitation, filtration, reverse osmosis, adsorption, wet oxidation, ozonation and other advanced oxidation processes (Pokhrel and Viraraghavan, 2004; Patel et al., 2021). However, these physicochemical wastewater treating processes are not cost-effective in large-scale operations and generally generate large amounts of toxic sludge as secondary pollutants (Kumar et al., 2022). Compared with physicochemical ways, biological methods for wastewater treatment are considered to be of cost–benefit, eco-friendly, and suitable for the reduction of BOD and COD from effluents (Hossain and Ismail, 2015). To date, many ligninolytic microbes and their enzymes as a single-step treatment or in combination with other physical and/or chemical methods, have been tried in the treatment of paper and pulp mill effluent (Chaudhry and Paliwal, 2018). A lignin peroxidase-producing bacterium *Serratia liquefaciens*, isolated from effluent contaminated soil, was used in the bioremediation of pulp and paper mill effluent (Haq et al., 2016). Chandra and Singh (2012) used a bacterial consortium of *Pseudochrobactrum glaciale*, *Providencia rettgeri,* and *Pantoea* sp. to treat pulp and paper mill effluent and found that ligninolytic enzymes including laccase (Lac), lignin peroxidase (LiP), and manganese peroxidase (MnP) were produced during the bioremediation of effluent. Among these enzymes generated by ligninolytic microbes, heme peroxidases such as lignin peroxidase (LiP), manganese peroxidase (MnP), and versatile peroxidase (VP) are well known and play a key role in lignin degradation (Falade et al., 2016). More importantly, these peroxidases are not stringently selective of substrates and their substrate profile is further broadened with the aid of small molecule mediators (Wang et al., 2018). The wide substrate specificity enables them to degrade and remove various environmental pollutants or xenobiotic compounds, raising interest of utilizing these heme peroxidases for bioremediation of industrial effluents (da Silva Vilar et al., 2022).

Dye-decolorizing peroxidase (EC. 1.11.1.19), belongs to a novel superfamily of heme peroxidases (DyP-type peroxidase superfamily; Yang et al., 2019). Although DyPs share no homology with classic fungi heme peroxidases, such as MnP, VP, and LiP aforementioned, they exhibit similar oxidation properties to these peroxidases (Hofrichter et al., 2010; Brissos et al., 2017), enabling DyPs to be more attractive for the bio-treatment of industrial wastewater (Ren et al., 2022). Although there have some studies regarding the potential of DyPs in degradation of phenolic and nonphenolic lignin model dimer, to date, there were no reports related to the application of DyPs in the remediation of paper and pulp mill effluent, in which lignin is considered as the major pollutant. The DyP from *Bacillus amyloliquefaciens* (BaDyP) has been previously cloned and heterogeneously expressed in *Escherichia coli* in our laboratory. The recombinant BaDyP exhibits a broad tolerance to pH and temperature than other ligninolytic heme peroxidases, which means that there will be a great application potential for BaDyP in the bioremediation of industry effluent (Yang et al., 2019; Zhang et al., 2021). Herein, we aimed to investigate the substrate profile of BaDyP and to evaluate the application potential of BaDyP in the bioremediation of pulp and paper mill effluent in order to explore a new biocatalyst for the bioremediation of pulp and paper mill effluent.

## Materials and methods

### Chemicals

2, 2′-azino-bis (3-ethylbenzothiazoline-6-sulfonic acid) (ABTS); was of 98% purity and purchased from Solarbio (Beijing, China). Isopropyl β-D-1-thiogalactopyranoside (IPTG), Reactive Blue 19 (RB19), Reactive Black 5 (RB5), lignin model dimer, and guaiacol were purchased from Sigma-Aldrich (Shanghai, China). A sample of effluent from the wastewater treatment workshop of paper mill manufacturer named Quanlinbense Co. Ltd. (Shandong, China), was collected in a pre-cleaned 5-L polypropylene container. Before experiments, the wastewater was filtered and stored at 4°C. The raw wastewater had the following chemical parameters: dark, 31.0°C, COD 7,670 mg/l, pH 11.3, turbidity 227 NTU.

### Recombinant BaDyP

Cloning, expression, and purification of recombinant BaDyP were performed according to protocols described in our previous work (Yang et al., 2019). pET30a-efeb was transformed into *E. coli* BL21 (DE3). Recombinant BaDyP was induced with 0.8 mm IPTG at 16°C. After 12 h, cells were collected and treated by sonication. The recombinant BaDyP in the soluble fraction was extracted and purified with Ni-NTA sefinose resin kit (Shanghai Sangon Biotech Co., Ltd., China).

The activity of BaDyP was assayed according to the oxidation of ABTS. To 1.9 ml tartrate buffer (pH 3, 50 mm) containing ABTS (10 mm), a volume of 0.1 ml BaDyP solution was added. The reaction was initiated by the addition of H_2_O_2_ (50 mm). The reaction was kept for 5 min at 25°C and the oxidation of ABTS to its cation radical was measured using a U-1600 UV–vis spectrometer (MAPADA, China) at 420 nm. One activity unit (U) of BaDyP was defined as the amount of enzyme required to oxidize 1 mmol of ABTS per minute.

### Substrate profile of BaDyP

The peroxidase activities of BaDyP against various substrates were determined by monitoring the absorbance change at the maximum absorption wavelength of each substrate or product: reactive black 5 (RB5), 597 nm; reactive blue 19 (RB19), 595 nm; MnSO_4_, 240 nm; veratryl alcohol, 310 nm; guaiacol, 470 nm.

BaDyP activity against lignin model dimer guaiacylglycerol-β-guaiacyl ether (GGE) was determined by Gas Chromatograph-Mass Spectrometer (GC–MS). In reaction system, 0.1 mm GGE in 100 μl of acetone solution was used as substrate, replacing ABTS. The reaction was initiated by adding 50 mm of H_2_O_2_ and incubated at 25°C and 180 r/min for 10 h. The reaction was quenched with 10 ml ethyl acetate. Then the organic layer was dried over Na_2_SO_4_ and concentrated under vacuum. The residues were silylated with trimethyl silyl (TMS), and the silylated derivatives were analyzed by GC–MS (GCMS-QP2010SE) with a HP-5 capillary column (50 m × 0.32 mm × 0.25 μm; NIST 11) as described by Yang et al. (2019). GGE-derived compounds were identified by comparing their mass spectra with those of the NIST library and by comparing the retention time with those of authentic compounds. The treatment with H_2_O_2_ was set as control.

### Kinetic analysis of BaDyP using ABTS and veratryl alcohol as substrates

In a 4 ml standard cuvette, a volume of 1.9 ml tartrate buffer (pH 3, 50 mm) containing substrate (ABTS and veratryl alcohol) and BaDyP, and hydrogen peroxide (100 μl, 40 mm) were mixed in the order stated, and the absorbance was monitored for 5 min at the corresponding maximum absorption wavelength (420 nm for ABTS and 310 nm for veratryl alcohol). Substrate concentrations were then varied for ABTS (final concentration of 1.25–10 mm) and veratryl alcohol (final concentration of 0.05–0.25 mm), with the total volume kept at 2.0 ml.

Michaelis constant (*K_m_*) and maximum velocity (*V*_max_) values were calculated from Lineweaver–Burk plot.

### COD and color removal of paper mill effluents treated by BaDyP

#### Treatment of paper mill effluents

The reactions proceeded in a 10 ml centrifuge tube containing 1.9 ml of paper mill effluents diluted with tartrate buffer (100 mm, pH 3), 100 μl BaDyP and 50 mm H_2_O_2_. The diluted paper mill effluents treated by H_2_O_2_ were set as a control.

To determine the optimum pH for removal of COD and chromaticity from effluent mediated by BaDyP, 100 mm of tartrate buffer for pH 2.5-pH 6.0 were used at 25°C. For the temperature optimum, 100 mm of tartrate buffer for optimum pH was used. The incubation time optimum was assayed at the optimum pH and temperature.

#### Chemical analysis

The performance of BaDyP on effluent treatment was assessed by the removal of COD and chromaticity, as well as lignin degradation. COD was measured according to the method reported previously (Saratale et al., 2018). Chromaticity was determined platinum-cobalt method reported by Hongve and Akesson (1996). Lignin content was determined by using acetyl bromide spectrophotometric method according to the lignin content detection kit (Solarbio, Beijing, China; Fukushima et al., 2021). Removal (degradation) rate (%) was defined as the percent decrease in the volume measured after the treatment as compared with that before the treatment [(volume before treatment –volume after treatment) /volume before treatment×100].

### Detoxification of paper mill effluents

Phytotoxicity of paper mill effluents treated and untreated was evaluated with *Vigna unguiculata* (Chow et al., 2018), while cytotoxicity of effluent was assessed with two bacteria strains including *Escherichia coli* and *Bacillus subtilis* (Kang et al., 2009). Seeds of *Vigna unguiculata* were pre-soaked in warm distilled water for 12 h before germination, and then were used to test the toxicity of effluents. In germination test, these pre-soaked seeds were placed in separate Petri dishes with three layers of filter papers and incubated in 28°C incubator. These seeds were moistened twice daily with 2 ml of treated and untreated effluents, respectively. After 3 days, the physiological indexes of seeds were evaluated, including germination percentage, radicle length. Triplicate samples of 10 seeds were used for each test (Bharagava et al., 2018). Cytotoxicity of effluent samples on the two strains was assessed *via* agar well-diffusion method. The inhibition zone diameters (mm) were measured after an incubation of 24 h at 37°C. Distilled water was used as a control.

## Results and discussion

### Cloning, expression, and purification of recombinant BaDyP

**Figure 1:**
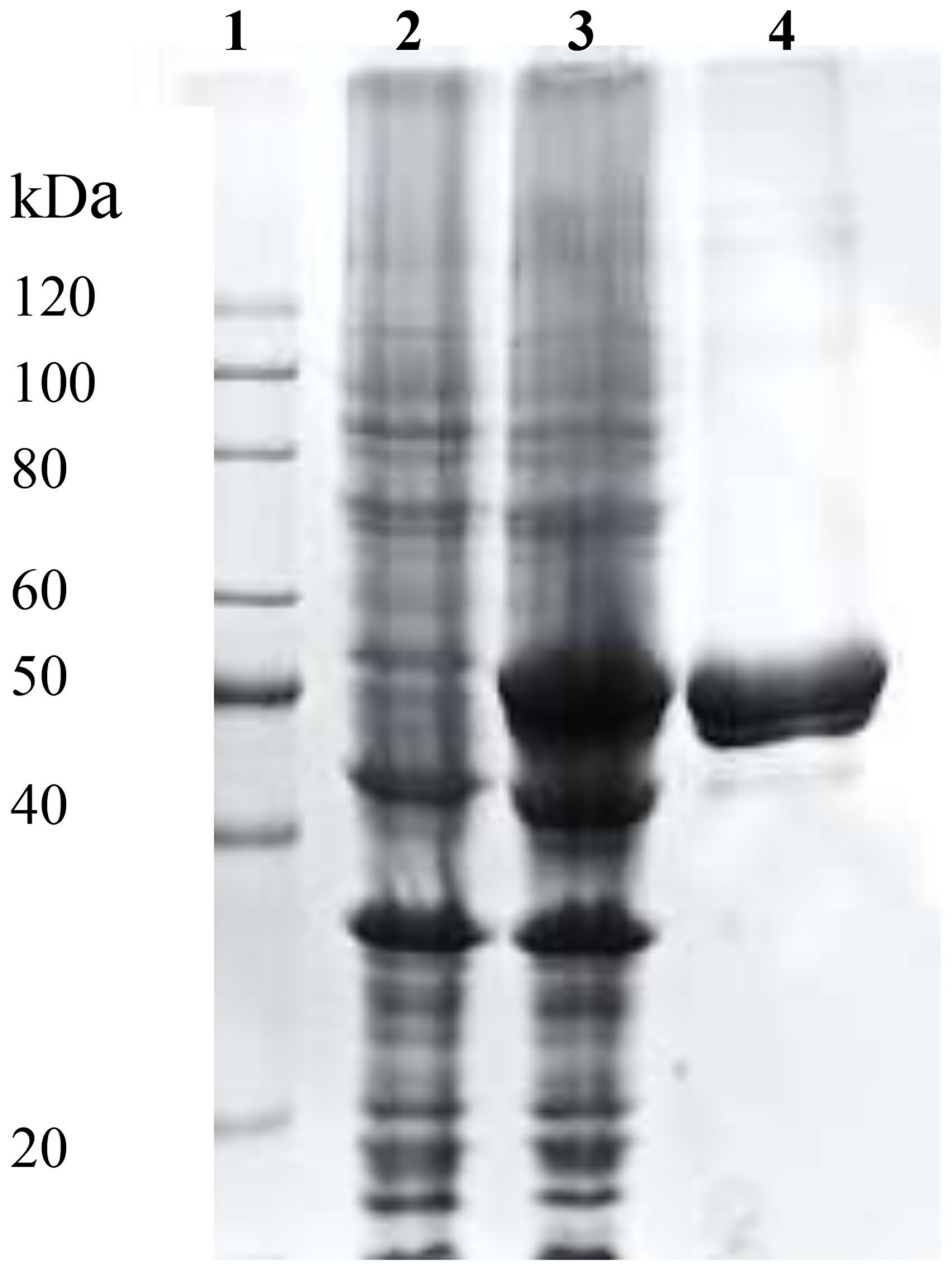
SDS-PAGE analysis of recombinant BaDyP expression and purification. Lane1, Marker; Lanes 2 and 3, Whole cell extract from *Escherichia coli* with an indicated expression vector without (-IPTG) and with (+IPTG) expression induction, respectively; Lane 4, Purified BaDyP eluted from Ni column with 80 mm imidazole.

The complete DNA sequence of *efeB* gene encoding BaDyP contains 1,257 bp. SDS-PAGE analysis of the purified BaDyP showed a visible protein band with a molecular weight of 45.8 kDa (Figure 1), which was consistent with the results achieved in our previous work (Yang et al., 2019).

### Substrate profile of BaDyP

**Figure 2:**
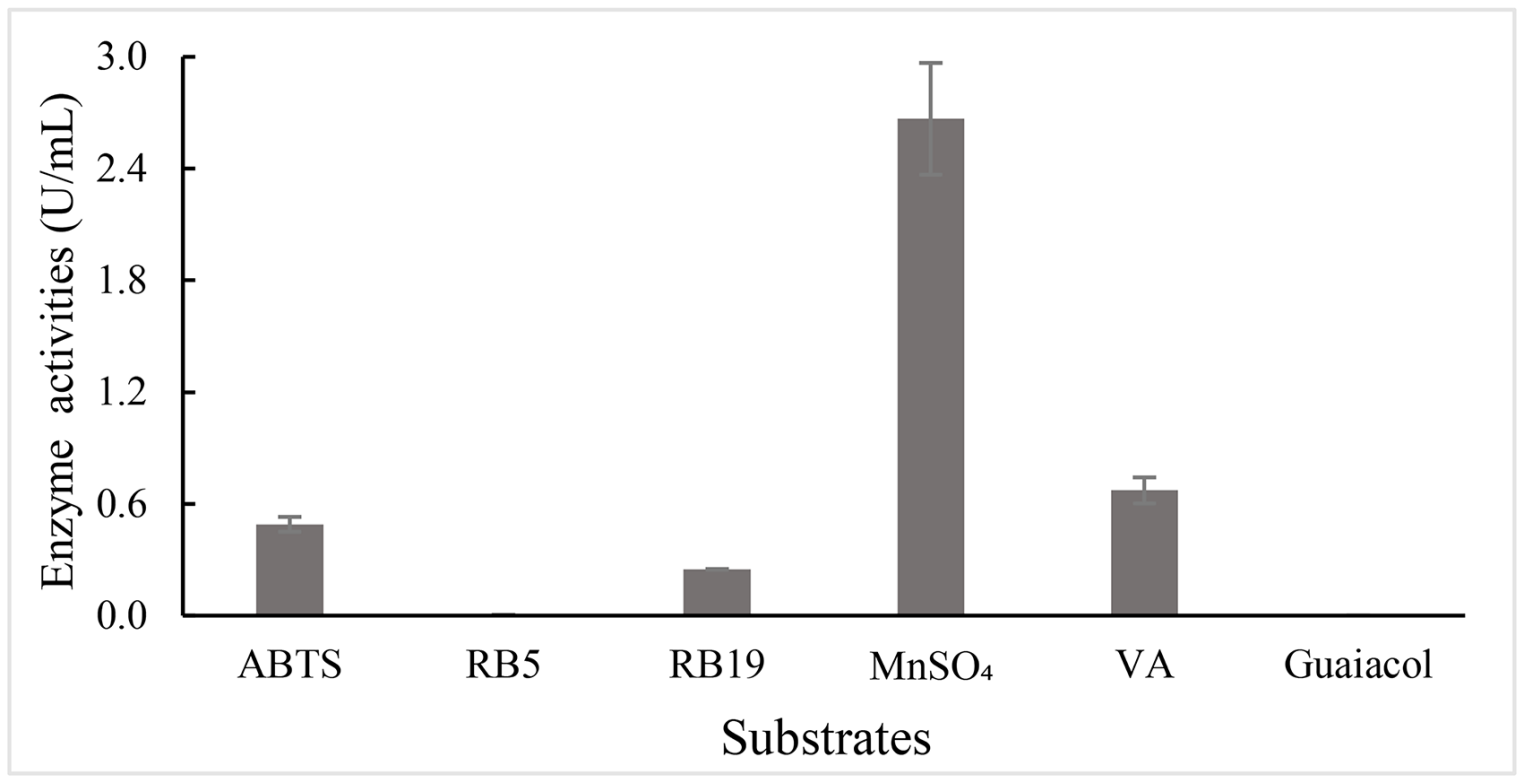
Specific activities of BaDyP using various substrates.

To investigate substrate spectrum of BaDyP, various substrates including common synthetic dyes for dye-decolorizing biocatalyst (azo dye Reactive Black 5 and anthraquinone dye Reactive Blue 19), organic compounds usually used as substrates of ligninolytic peroxidase (ABTS, Mn^2+^, guaiacol, and VA), and β-O-4 lignin dimer model (GGE) were tested at pH 3 and 25°C. BaDyP shows relatively good substrate breadth (Figure 2), in which BaDyP can oxidize various substrates, such as dyes and some substrates with relatively high redox potential (ABTS, VA, and Mn^2+^), but it cannot oxidize guaiacol into dimer or trimer like DypB from *Rhodococcus jostii* RHA1 (Ahmad et al., 2011). In addition, the decolorization activity of BaDyP for azo dye RB5 (0.043 U/ml) was much lower than that for anthraquinone dye RB19 (0.248 U/ml), which is similar to the oxidation character of DyP from the cyanobacterium *Anabaena* species and exact opposite to that of DyP from *Bacillus subtilis* (Ogola et al., 2009; Min et al., 2015).

**Figure 3:**
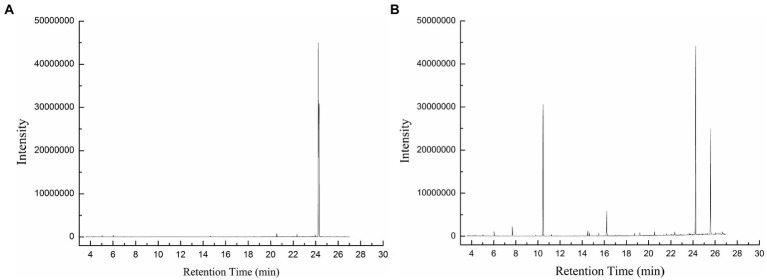
The GC–MS total ionic current (TIC) chromatogram of GGE and GGE-derived compounds. **(A)** GGE in control; **(B)** GGE-derived compounds.

At pH 3 and 25°C, BaDyP not only exhibited high oxidation activity to various substrates, but also successfully decomposed phenolic lignin dimer GGE (Table 1; Figure 3). In GC–MS analysis of GGE treated with H_2_O_2_ in control group, GGE was detected at R.T. 24.30 min (Figure 3A). However, after 10 h of treatment with BaDyP/H_2_O_2_, degradation products of GGE and their trimethylsilylation derivatives, such as guaiacol (1 in Figure 3B; Table 1), guaiacol-TMS (2 in Figure 3B; Table 1), vanillyl alcohol di-TMS (3 in Figure 3B; Table 1), 2-methoxy-4-(1,3-hydroxy-2-propenyl) phenol di-TMS (4 in Figure 3B; Table 1), and vanillin-TMS (5 in Figure 3B; Table 1) were observed. Although the signal at retention time 24.23 min is close to that of GGE in the total ion chromatogram, the mass spectrum of the component was identical to 2-methox-4-(1, 3-hydroxy-2-prpenyl) phenol di-TMS, not GGE, indicating that GGE was completely degraded by BaDyP. DypB from *Rhodococcus jostii* RHA1 was reported to decompose lignin model dimer GGE into guaiacol and vanillin by cleavage of C_α_-C_β_, and oxidize furtherly guaiacol into guaiacol trimer. In this study, both vanillin and guaiacol were observed in the degradation derivatives of GGE, indicating C_α_-C_β_ bond cleavage was also happened in the degradation of GGE by BaDyP. However, there is a little difference between BaDyP and DyPB, of which BaDyP cannot oxidize guaiacol into trimer, which is consistent with the result in the analysis of oxidation activity against guaiacol. In addition, intermediates of vanillyl alcohol and 2-methoxy-4-(1, 3-hydroxy-2-propenyl) phenol were generated, indicating that fission of β-O-4 bond in GGE might be happened prior to the cleavage of C_α_-C_β_ in the GGE degradation mediated by BaDyP.

**Table 1:** GGE and its degradation derivatives identified by GC–MS.

Entry	Retention time (min)	Compounds	Control	With BaDyP
1	7.65	Guaiacol	/	+
2	10.40	Guaiacol, trimethylsilyl ether	/	+
3	16.20	Vanillyl alcohol, bis (trimethylsilyl) derivate	/	+
4	24.23	Phenol, 2-methoxy-4-(1,3-hydroxy-2-propenyl), bis (trimethylsilyl) derivate	/	+
5	25.57	Vanillin, trimethylsilyl ether	/	+
6	24.30	GGE, trimethylsilyl derivate	+	/

“+” compound observed. “/” compound not observed.

### Kinetic constants of BaDyP

**Figure 4:**
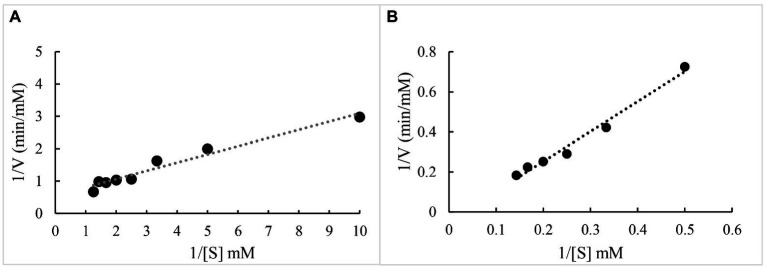
Linewaver-Burk plot of BaDyP for the oxidation of ABTS and veratryl alcohol. **(A)** ABTS; **(B)** veratryl alcohol.

The kinetics of oxidation of ABTS/veratryl alcohol by BaDyP were studied and the Lineweaver–Burk plot was shown in Figure 4. The kinetic constant *K_m_* of BaDyP for ABTS was 2.19 mm and *V_max_* was 1.8 mm/min (Figure 4A), while *K_m_* of BaDyP for veratryl alcohol was 0.07 mm and *V_max_* was found to be 14.12 mm/min (Figure 4B). In present and previous studies, two types of substrate-interaction sites in the oxidation process of DyPs against substrates have been well elaborated. DyPs can bind small organic molecules like imidazole in its heme cavity (Strittmatter et al., 2015). Regarding bulky substrates, DyPs withdraw electrons from bulky substrates using long-range electron transfer routes from surface-exposed tryptophanyl or tyrosyl radicals to the heme, because the narrow heme-access channel of DyP clearly prevents on-site oxidation at the heme molecule (Brissos et al., 2017). The different *K_m_* of BaDyP for ABTS and veratryl alcohol in this study, indicating that the binding affinity of BaDyP with veratryl alcohol is higher than that of BaDyP with ABTS, might be caused by different types of substrate-interaction sites due to different molecular sizes of the two substrates.

### Bioremediation of paper and pulp mill effluents by BaDyP

DyPs can use H_2_O_2_ as the final electron acceptor to catalyze the oxidation of a wild range of compounds, from high redox synthetic dyes and aromatic sulfides to iron, manganese ions, phenolic and nonphenolic lignin units, which endow them with application potential in many industries (Brissos et al., 2017). In this study, BaDyP was also confirmed to exhibit similar oxidative activity to other DyPs. On the other side, paper and pulp mill effluents are closely associated with degraded and dissolved lignin which caused serious pollution, toxic effects, and high concentration of color. Therefore, to evaluate the potential of BaDyP in the bioremediation of paper and pulp industrial effluent maybe explore a high efficient biocatalyst for the treatment of industrial wastewater.

#### Effect of different concentration of effluent on COD and color removal

**Figure 5:**
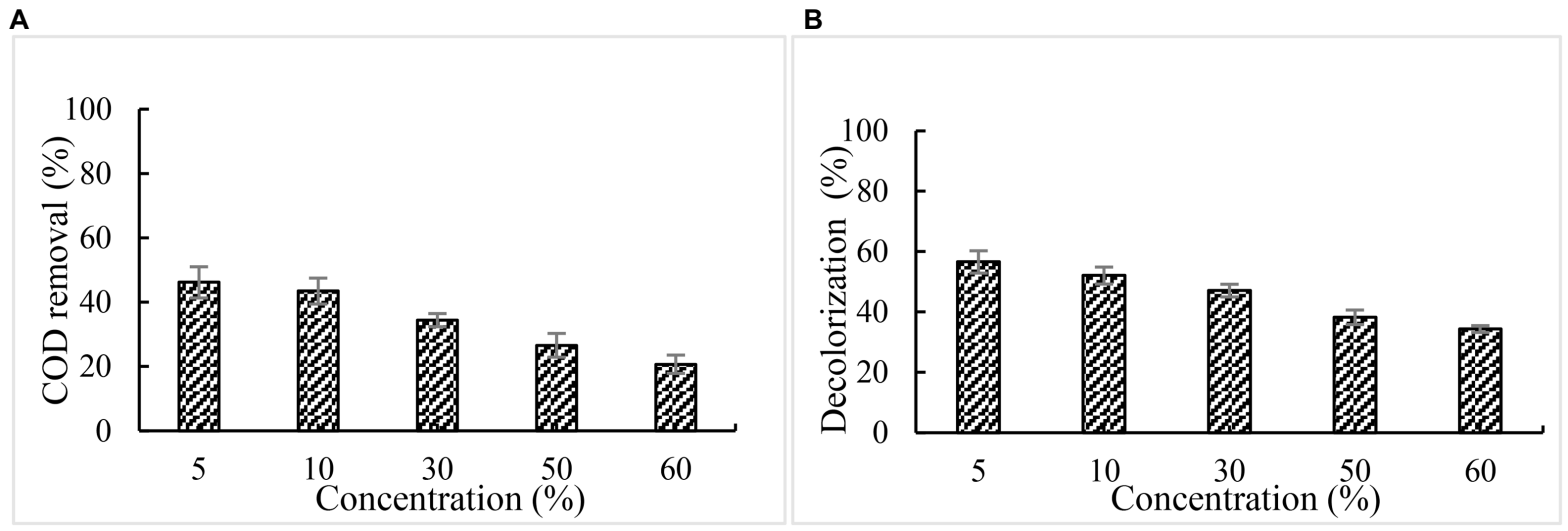
Effect of effluent concentration on COD and color removal. **(A)** COD; **(B)** chromaticity.

The treatment of paper mill effluents with BaDyP/H_2_O_2_ was conducted at 30°C and pH 3. After incubation of 72 h, the COD and chromaticity of paper and pulp industrial effluent at different concentration got decreased to varying degrees (Figure 5). Among these different concentrations of effluent, a significant decrease was observed in the treatment of 5% effluent with BaDyP/H_2_O_2_, in which COD and color removal were 46.4 and 56.7%, respectively.

#### Effect of pH on COD and color removal

**Figure 6:**
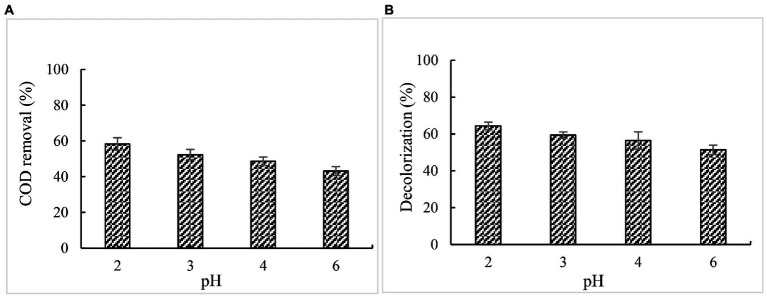
Effect of pH on COD and color removal. **(A)** COD; **(B)** chromaticity.

According to the results reported previously, the optimum pH for DyPs varied depending on the substrates, *e. g.* DyP from *Irpex lacteus* exhibited substrate-dependent optimum pH, of which the optimum pH for veratryl alcohol is 2.0, for 2, 6-dimethoxyphenol and reactive black 5 is pH 3.0, and for reactive blue is pH 4.0 (Salvachúa et al., 2013). Min et al. reported that DyP from *Bacillus subtilis* (BsDyP) showed substrate-dependent optimum pH. In our previous study, the optimum BaDyP activity towards veratryl alcohol was at pH 4.0. Whereas, when 5% paper and pulp mill effluent was used to determine the optimum pH of BaDyP for the removal COD and chromaticity of effluent, the optimum pH was 2.0 which is different from that for veratryl alcohol, indicating that BaDyP exhibits substrate-dependent optimum pH like BsDyP and DyP from *Irpex lacteus*. At pH 2.0, the COD removal rate was 58.4%, and the decolorization rate was 64.4%. With pH increasing, COD and color removal decreased (Figure 6).

#### Effect of temperature on COD and color removal

**Figure 7:**
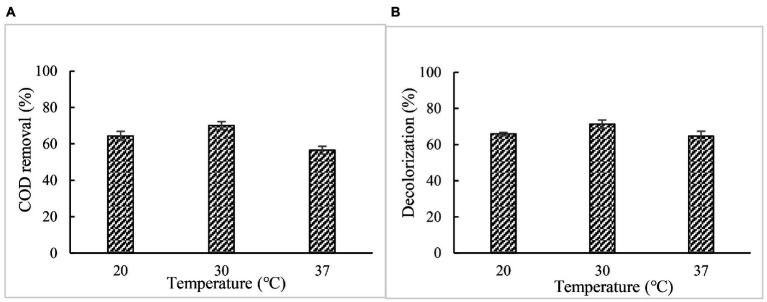
Effect of temperature on COD and color removal. **(A)** COD; **(B)** chromaticity.

When 5% paper and pulp mill effluent was treated with BaDyP/H_2_O_2_ at pH 2 and at different temperature for 72 h, respectively, the optimal temperature for COD and color removal were found at 30°C, at which BaDyP exhibited optimum oxidation activity to veratryl alcohol. Based on the result we concluded that BaDyP showed substrate-independent optimum temperature, which is different from BsDyP which was reported to exhibit substrate-dependent optimum pH and temperature by Min et al. (2015). The highest COD and color removal efficiency were achieved with 70.0 and 71.4% (Figure 7).

#### Effect of incubation time on COD and color removal

**Figure 8:**
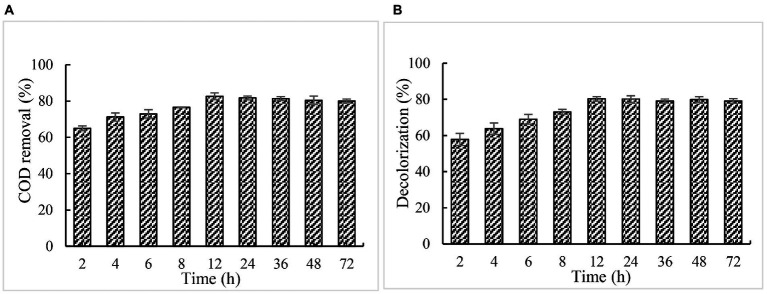
Effect of incubation time on COD and color removal. **(A)** COD; **(B)** chromaticity.

When 5% of paper mill effluents was treated at the optimal conditions of pH 2 and 30°C for 12 h, the maximum COD removal and decolorization of paper and pulp mill effluent were achieved with 82.7 and 80.2%, respectively (Figure 8). Under this same condition, lignin degradation of effluent reached 78.20%. Further prolonging the reaction time, no obvious increase, and even a slight decrease in COD removal, decolorization, and lignin degradation were observed.

### Detoxification of paper mill effluents treated by BaDyP

The ultimate goal of bioremediation was to reduce the toxicity of effluent. Therefore, detoxification of effluent is a key indicator to evaluate the performance of ligninolytic microbes and their enzymes in the bioremediation of pulp and paper mill effluent. In this study, phytotoxicity and cytotoxicity of paper mill effluents treated and untreated were evaluated and the results are shown in Table 2. The seeds grown in untreated effluent showed a significant decrease in germination rate of *Vigna unguiculata* from 92.3% (distilled water) to 66.67%, and in radicle length from 1.57 cm (distilled water) to 0.68 cm, respectively, indicating the high toxicity of the untreated effluent in nature. However, the germination rate and radicle length of V*igna unguiculata* seeds grown in BaDyP-treated effluent were 90% and 1.26 cm, respectively, which increased by 35.0 and 85.3% compared to the result of untreated effluent. In addition, after treatment by BaDyP, a significant decrease in inhibition of paper and pulp mill effluent on *E. coli* and *B. subtilis* was observed, in which antibacterial activity of effluent against *B. subtilis* and *E. coli* with inhibition zone diameter of 12.2 mm and 10.5 mm to 0.0 mm, respectively. From all these results, it can be confirmed that an outstanding detoxification has been achieved in the treatment of paper and pulp mill effluent mediated by BaDyP.

**Table 2:** Toxicity of industrial wastewater before and after treatment by BaDyP.

		*Vigna unguiculata*		Diameter of inhibition zone (mm)	
		Germination %	Radicle length(cm)	*B. subtilis*	*E. coli*
Distilled water	Control	92.30 ± 3.27	1.57 ± 0.25	—	—
5% Paper and pulp mill effluent	Treated	90.00 ± 1.76	1.26 ± 0.31	0.0	0.0
	Untreated	66.67 ± 2.35	0.68 ± 0.17	12.2	10.5

It is well known that, in addition to their peroxidatic activities, heme containing peroxidases possess an oxidase activity by which electrons can be transferred from reducing substrates to O_2_, resulting in the formation of superoxide radical anions O_2_^•-^ in the catalytic cycle of peroxidase (Yokota and Yamazaki, 1965). Therefore, it is easy to conclude that dissolved oxygen in effluent can easily achieve one electron to generate O_2_^•-^ during the treatment of pulp and paper mill effluent mediated by BaDyP. Based on the production of O_2_^•-^, hydroxyl radicals (^•^OH) with high redox potential could be subsequently generated *via* the Habere-Weiss reaction of O_2_^•−^ with H_2_O_2_ (H_2_O_2_ + O_2_^•−^ → ^•^ OH + OH^−^ + O_2_; Ju et al., 2013). As we all know, the toxicity of pulp and paper mill effluent comes primarily from lignin and its chlorinated derivatives (*e. g.* various lignin-derived phenolics). In this study, it is clear that BaDyP can degrade lignin dimer, but cannot oxidize guaiacol, which means a low performance of BaDyP for the removal of lignin-derived phenolic compounds. However, high detoxification performance of BaDyP for the treatment of pulp and paper mill effluent was achieved in this study. Thus, the high detoxification performance is undoubtedly ascribed to the combinative effect of oxidative power of BaDyP and ^•^OH generated in the oxidation cycle of BaDyP. The similar result has been reported by Datta et al., that ligninolytic peroxidases (*e. g.* LiP, MnP, and VP) can directly attack lignin, and can also degrade lignin and even mineralize the insoluble lignin through low molecular weight free radicals such as ^•^OH (Datta et al., 2017). Additionally, hydroxyl radical, by far the most powerful oxidizing radical species and non-selective oxidizing agent (*E*°= 2.80 V) can initiate polymerization of many organics. This might be the reason the maximum COD and color removal was achieved at 12 h of inoculation and then decreased along with the time extending.

It is well known that ligninolytic enzyme undoubtedly possesses the potential to remove organic load from paper and pulp mill effluent and to improve the quality of wastewater. However, there has been few reports related to the application of these enzymes in bioremediation of real paper industrial wastewater. Oppositely, there have been a great number of studies regarding the application of ligninolytic enzyme-producing microorganisms in the bioremediation of paper industrial effluent. For example, a laccase-producing *Paenibacillus* sp. strain LD-1 (JX499920) isolated from contaminated soil sample effectively remove 68% color, 54% lignin, 86% phenol, 83% BOD and 78% COD from paper and pulp mill effluent after 144 h of treatment at 34°C (Raj et al., 2014). A ligninolytic *Bacillus cereus* was reported by Kumar et al. (2022) to lead to significant reduction of pollutants (KL-72.5%, color-62.0%, COD-45.05%) in real paper and pulp mill effluent after 5 day of treatment. When a lignin peroxidases-producing *Serratia liquefaciens* strain was used to treat pulp and paper mill effluent at 30°C, pH 7.6, and 120 rpm for 144 h, the reduction of COD, color, and phenolic content by 84, 72, and 95% were achieved after 144 h of treatment, respectively (Haq et al., 2016, 2017). When two strains of *Bacillus cereus* (ITRC-S6) and *Serratia marcescens* (ITRC-S7) were used for the treatment of pulp and paper mill effluent, the highest reduction in color (62%), lignin (54%), COD (90%) and total phenol (90%) was obtained within 168 h of incubation (Chandra et al., 2009). Chandra et al. (2010) also used bacterial consortium comprising *Serratia marcescens* (GU193982), *Citrobacter* sp. (HQ873619), and *Klebsiella pneumoniae* (GU193983) to treat black liquor from pulp and paper mill, and found that the developed bacterial consortium was efficient for the reduction of COD, BOD, and color up to 83, 74, and 85%, respectively. Compared with these microorganisms, BaDyP reported in the present study exhibited higher color and COD removal efficiency, of which a significant reduction of COD (82.7%), color (80.2%), and lignin (78.2%), and detoxification was achieved within 12 h (Table 3). Additionally, BaDyP-mediated treatment can convert or transform toxic pollutants into non/less-toxic compounds rapidly, and have no such limitations involved in the bioremediation of effluent by bacteria, e.g., requiring long periods of time to grow and have an effect, suitable environmental and growth conditions (e.g., pH, temperature, aerobic or anaerobic condition and level of toxic compound), and adequate levels of nutrients (carbon and nitrogen source), etc. Horseradish peroxidase, which is often termed the classical plant heme peroxidase, used to be tried to treat pulp and paper mill effluent (Peralta-Zamora et al., 1998), whereas the performance of horseradish peroxidase was not better than BaDyP in the treatment of pulp and paper mill effluent (Table 3).

**Table 3:** Comparison of BaDyP and other biological approaches for the treatment of pulp and paper wastewater.

Enzymes or cells	Reaction temperature	Reaction time	Dye removal (%)	Reference
BaDyP	30°C	12 h	Color removal 80.2%, COD reduction 82.7% and lignin reduction 78.2%	This study
Laccase-producing *Paenibacillus* sp. strain LD-1	34°C	144 h	Color removal 68%, lignin reduction 54%, COD reduction 78% and phenol reduction 86%	Raj et al. (2014)
Ligninolytic *Bacillus cereus*	/	5 d	Color removal 62.0%, COD reduction 45.05% and KL reduction 72.5%	Kumar et al. (2022)
Lignin peroxidases producing *Serratia liquefaciens* strain	30°C	144 h	Color removal 72%, phenolic content reduction 95% and COD removal 84%	Haq et al. (2017)
*Bacillus cereus* and *Serratia marcescens*	30°C	168 h	The removal of color, lignin, BOD, COD, and total phenols were 62, 54, 70, 90, and 90%, respectively	Chandra et al. (2009)
Bacterial consortium comprising *Serratia marcescens, Citrobacter* sp., and *Klebsiella pneumoniae*	35°C	196 h	The reduction of COD, BOD, and color up to 83, 74, and 85%, respectively.	Chandra et al. (2009)
Immobilized horseradish peroxidase on IRA-400 resin	/	160 min	Color removal 52.8%	Peralta-Zamora et al. (1998)

## Conclusion

Enzyme-mediated bioremediation is a promising and efficient biological method for industrial effluent treatment. However, the application of enzyme in real paper and pulp mill effluent is poorly studied. Thus, application potential of BaDyP in treatment of paper and pulp mill effluent were evaluated in this work. The results revealed that BaDyP exhibited broad substrate spectrum and using BaDyP as a catalyst for the treatment of pulp and paper mill effluent could be operationally and economically, and high efficient in the removal of COD, color, and lignin. Up to 82.7% COD reduction, 80.2% of color removal, and 78.20% lignin degradation were obtained at pH 2, 30°C and inoculation time of 12 h in treatment system of 5% pulp and paper mill effluent mediated by BaDyP. Among these factors affecting the performance of BaDyP in the treatment of paper and pulp mill effluent, temperature was the most influential parameter for COD removal, while effluent concentration was the most influential parameter for decolorization. Additionally, it should be noted that there are several limitations of BaDyP-mediated bioremediation for the large-scale application, such as the production cost of enzyme, and its stability and activity against pH, temperature, and other harsh environmental conditions. Despite such limitations that need to be addressed, BaDyP still appeared to be an environmentally friendly, technically favorable, and effective biocatalyst with potential for use in large-scale bioremediation systems of pulp and paper wastewater.

## Data availability statement

The original contributions presented in the study are included in the article/supplementary material, further inquiries can be directed to the corresponding authors.

## Author contributions

JR: methodology and writing original draft. XL: methodology. WZ: methodology and formal analysis. ZL: formal analysis. QW: data curation. SL: review and editing. SW: resources and formal analysis. HL: conceptualization, supervision, and review and editing. All authors contributed to the article and approved the submitted version.

## Funding

This work was supported by the Science and Technology Planning Project of Hebei Province (20322909D), the Natural Science Foundation of Hebei Province (C2020204149), and the Central Government-guided Local Science and Technology Development Fund Project of Hebei Province (226Z2902G).

## Conflict of interest

The authors declare that the research was conducted in the absence of any commercial or financial relationships that could be construed as a potential conflict of interest.

## Publisher’s note

All claims expressed in this article are solely those of the authors and do not necessarily represent those of their affiliated organizations, or those of the publisher, the editors and the reviewers. Any product that may be evaluated in this article, or claim that may be made by its manufacturer, is not guaranteed or endorsed by the publisher.
